# F220. THREAT ANTICIPATION AND NEGATIVE AFFECT IN EARLY PSYCHOSIS

**DOI:** 10.1093/schbul/sby017.751

**Published:** 2018-04-01

**Authors:** UnYoung Chavez-Baldini, Inez Myin-Germeys, Charlotte Gayer-Anderson, Matthew Kempton, Lucia Valmaggia, Philip McGuire, Til Wykes, Craig Morgan, Ulrich Reininghaus

**Affiliations:** 1 Academic Hospital Maastricht; 2 KU Leuven; 3 Institute of Psychiatry, Psychology & Neuroscience, King’s College London; 4 Institute of Psychiatry Psychology and Neuroscience, King’s College London, South London, Maudsley NHS Foundation Trust; 5 Maastricht University

## Abstract

**Background:**

Increasingly, evidence points to the involvement of cognitive and affective processes in psychotic disorders. To determine the interplay of mechanisms involved in the development and maintenance of psychosis, these pathways must be studied in different stages of psychosis, such as early psychosis. Previous research, however, mostly uses cross-sectional data, and there remains a need to extend research to include timeseries and longitudinal models to investigate the direction of the relationship between these processes and psychotic experiences.

**Methods:**

Lagged multilevel moderated mediation models were used to analyze the experience sampling method (ESM) data of 53 controls, 46 participants with at-risk mental state (ARMS) for psychosis, and 51 participants with first-episode psychosis (FEP) to investigate the direction of effect between threat anticipation, negative affect, and psychotic experiences. Furthermore, specific affect symptoms (i.e., anxiety and insecurity, separately) and psychotic experiences (i.e., paranoia and visual and auditory hallucinations, separately) were analyzed.

**Results:**

The effect of threat anticipation (t0) on psychotic experiences (t1) was mediated by negative affect for ARMS participants and controls. Threat anticipation (t0) had a direct effect on psychotic experiences (t1) and psychotic experiences (t0) had a direct effect on threat anticipation (t1) for FEP participants. The relationship between threat anticipation (t0) and paranoia (t1) was mediated by anxiety for FEP participants and controls and mediated by insecurity for ARMS participants. Threat anticipation (t0) had a direct effect on auditory and visual hallucinations (t1) for FEP participants, and there was a direct effect of visual hallucinations (t0) on threat anticipation (t1) for ARMS participants.

**Discussion:**

The findings demonstrate that threat anticipation leads to psychotic experiences, including paranoia and hallucinations, and affective disturbances mediate some of the relationships. However, there was inadequate evidence for psychotic experiences, paranoia, and hallucinations leading to threat anticipation. Together, these results provide insight into the direction of cognitive and affective processes that develop and maintain psychotic experiences in early psychosis.

